# Endotoxin-Induced Tryptophan Degradation along the Kynurenine Pathway: The Role of Indolamine 2,3-Dioxygenase and Aryl Hydrocarbon Receptor-Mediated Immunosuppressive Effects in Endotoxin Tolerance and Cancer and Its Implications for Immunoparalysis

**DOI:** 10.1155/2015/973548

**Published:** 2015-12-31

**Authors:** Elisa Wirthgen, Andreas Hoeflich

**Affiliations:** Institute of Genome Biology, Leibniz Institute for Farm Animal Biology, Germany

## Abstract

The degradation of tryptophan (TRP) along the kynurenine pathway plays a crucial role as a neuro- and immunomodulatory mechanism in response to inflammatory stimuli, such as lipopolysaccharides (LPS). In endotoxemia or sepsis, an enhanced activation of the rate-limiting enzyme indoleamine 2,3-dioxygenase (IDO) is associated with a higher mortality risk. It is assumed that IDO induced immunosuppressive effects provoke the development of a protracted compensatory hypoinflammatory phase up to a complete paralysis of the immune system, which is characterized by an endotoxin tolerance. However, the role of IDO activation in the development of life-threatening immunoparalysis is still poorly understood. Recent reports described the impact of inflammatory IDO activation and aryl hydrocarbon receptor- (AhR-) mediated pathways on the development of LPS tolerance and immune escape of cancer cells. These immunosuppressive mechanisms offer new insights for a better understanding of the development of cellular dysfunctions in immunoparalysis. This review provides a comprehensive update of significant biological functions of TRP metabolites along the kynurenine pathway and the complex regulation of LPS-induced IDO activation. In addition, the review focuses on the role of IDO-AhR-mediated immunosuppressive pathways in endotoxin tolerance and carcinogenesis revealing the significance of enhanced IDO activity for the establishment of life-threatening immunoparalysis in sepsis.

## 1. Introduction

Each day, animal organisms are exposed to a multitude of immunological stressors and pathogens, and they react with appropriate immune responses. For a successful host defense, a balance between pro- and anti-inflammatory parts of the immune response is essential. A loss of this balance leads either to an overreaction or to a suppression of immune response, both of which could be life-threatening situations. Within this regulation of immune response, the degradation of tryptophan (TRP) along the kynurenine pathway plays a crucial role. This pathway is a major link between the immune and nervous systems [1]. In the context of immune response, indoleamine 2,3-dioxygenase 1 (IDO1) is the rate-limiting enzyme for the degradation of tryptophan (TRP) along the kynurenine pathway to biologically active metabolites, such as kynurenine (KYN), kynurenic acid (KYNA), or quinolinic acid (QUIN), which have been shown to take part in diverse physiological and pathological processes [2]. IDO1 is activated by proinflammatory cytokines as a part of innate immunity and has different physiological functions with a highly cell type specific pattern of inducibility. The immunosuppressive function of IDO1 activation was shown for the first time in murine placenta [3]. The high expression of IDO1 in the placenta leads to a local suppression of maternal immune response and protects the fetus from rejection by its mother. Even in malignancies, it has been shown that an increased IDO1 activity promotes the development of an immune tolerance protecting the tumor against the immune response [4]. In patients with sepsis, an increased IDO1 activity provokes systemic immunosuppression and is associated with an increased risk for mortality, when it is used as a prognostic indicator for probability of survival [5, 6]. In addition to the immunological consequences, the modulation of TRP metabolism influences the nervous system and behavior. The activation of the kynurenine pathway in the brain, depletion of TRP, and generation of neurologically active metabolites are associated with diseases including schizophrenia [7], Alzheimer's disease [8], and depression [9, 10].

## 2. Tryptophan Metabolism as a Link between Nutrition and Immune and Nervous System

TRP as an essential amino acid is not synthesized by the animal organism. Therefore, dietary TRP is transported from the digestive tract through the portal vein to the liver where it is used for the synthesis of proteins [11]. In numerous cell types, approximately 95% of the dietary TRP is metabolized via the kynurenine pathway [2, 12]. For a long time it was thought that TRP is only completely oxidized to NAD^+^ (coenzyme) or to ATP (energy source) in liver cells, but it has been shown that this occurs also in extrahepatic cells (e.g., in astrocytes) [13]. In many cell types, the metabolites of the kynurenine pathway mediate mainly anti-inflammatory effects [14] whereby they act regulatory in response to proinflammatory stimuli. In cells of the nervous system KYNA, anthranilic acid (AA), 3-hydroxyanthranilic acid (3-HA), or QUIN modulate neurological functions. KYNA acts as an antagonist of the glutamate receptor and is therefore described as a neuroprotective metabolite, whereas QUIN, an agonist of the glutamate receptor, mediates neurotoxic effects [15]. In addition to its importance in the kynurenine pathway, the degradation of TRP to serotonin (5-HT) and to melatonin in cells of the pineal gland has important physiological functions. 5-HT acts both as a hormone and as a neurotransmitter. Melatonin acts as a neurohormone and is associated with the development of circadian rhythm and the sleep-wake cycle [16]. Because the expression of enzymes along the kynurenine pathway may differ substantially between specific cell types, a crude classification of four compartments was made [11]. The first compartment includes cells of somatic tissues, such as the lungs and intestines, where TRP is mainly degraded to KYN. In addition to the immunosuppressive effects of KYN, it has been shown that KYN acts as an endothelium-derived relaxing factor that is able to decrease blood pressure in hypertensive rats [17]. The second compartment contains cells of the immune system, such as dendritic cells or macrophages, which express many enzymes of the kynurenine pathway, exclusive of enzymes for the synthesis of the coenzyme nicotinamide adenine dinucleotide (NAD^+^), which is required for redox reactions in cells. Hepatic cells represent the third compartment and express all enzymes required for the total oxidation of TRP. In the fourth compartment, including cells of the central nervous system, TRP is specifically degraded to KYNA, but other neurologically active metabolites, such as quinolinic acid, are also generated. Additionally, KYNA has been found to be generated by macrophages [18]. Increased concentrations of KYNA and xanthurenic acid (3-Hydroxy KYNA, XA) were detected in the plasma of patients with type 2 diabetes, presumably due to chronic stress or the low-grade inflammation that are prominent risk factors for diabetes [19]. Thermochemical and kinetic data show that KYNA and XA are the best free-radical scavengers from the eight tested TRP metabolites [20], suggesting that the production is a regulatory mechanism to attenuate damage by the inflammation-induced production of reactive oxygen species. AA and 3-HA can act as antioxidants in certain chemical environments but, in the presence of iron(II), 3-HA exhibits prooxidant activity [21]. Patients suffering from neurological disorders as Huntington's disease or brain injury often showed decreased levels of 3-HA combined with increased levels of AA [22]. However, the biological importance of the 3-HA to AA ratio as either neurotoxic or neuroprotective mechanism is still discussed [22, 23].

**Figure 1:**
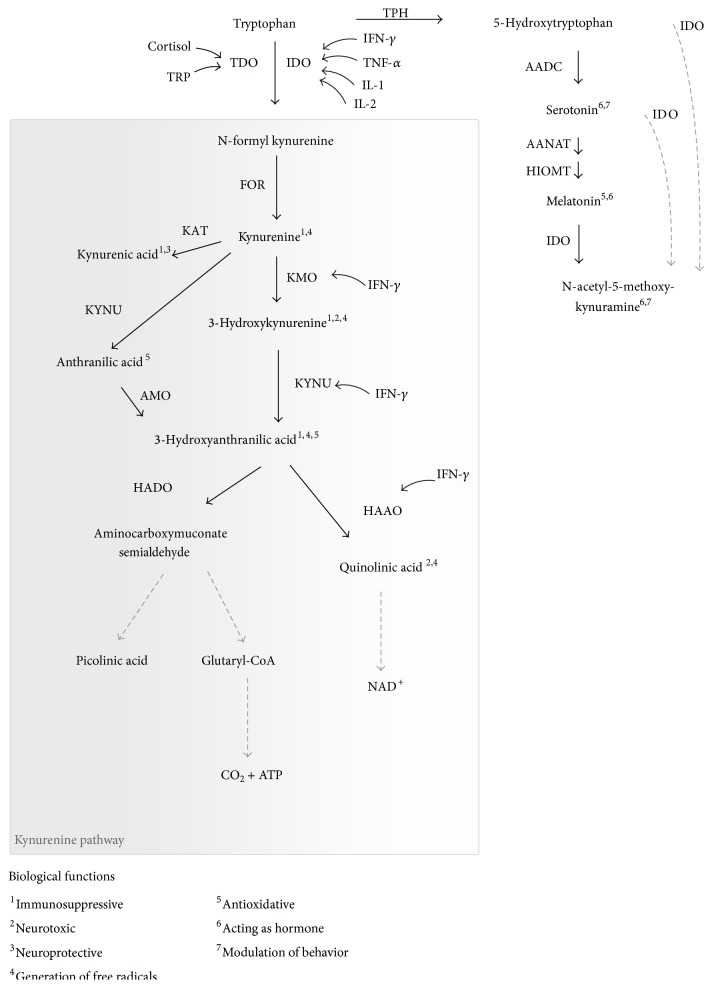
Main pathways of TRP degradation including enzymes and their potential stimuli (modified after [1, 4, 16, 35]). Superscripted numbers indicate described biological effects of TRP metabolites. Black arrows mark enzymatic reactions and dashed arrows include more than one catalytic reaction step. Enzymes: FOR = formamidase, IDO = indolamine 2,3-dioxygenase; TDO = tryptophan 2,3-dioxygenase, TPH = tryptophan hydroxylase; KAT = kynurenine aminotransferase, KMO = kynurenine 3-monooxygenase; KYNU = kynureninase, HADO = 3-hydroxyanthranilic acid dioxygenase; HAAO = 3-hydroxyanthranilic acid oxidase, AMO = anthranilate 3-monooxygenase, and AADC = aromatic L-amino acid decarboxylase; AANAT = N-acetyltransferase; HIOMT = hydroxy-O-methyltransferase.

One characteristic of TRP metabolism is that the rate-limiting step of the catalysis from TRP to KYN is generated by both the hepatic enzyme tryptophan 2,3-dioxygenase (TDO) and the ubiquitous expressed enzyme IDO1 [24, 25]. TDO is essential for homeostasis of TRP concentrations in organisms and has a lower affinity to TRP than IDO1. Its expression is activated mainly by increased plasma TRP concentrations but can also be activated by glucocorticoids and glucagon [26]. In contrast to TDO, IDO1 expression is specifically induced by inflammatory stimuli, such as the cytokines TNF-*α* or IFN-*γ*. IDO has a high affinity to L-tryptophan but will also catalyze the oxidation of other substrates, such as serotonin (5-HT) or melatonin [27]. It has been assumed that TDO is only expressed in the liver; however, studies in mice indicate that TDO is also expressed in the brain [28, 29]. In addition to IDO1 and TDO, IDO2 is described as the third rate-limiting enzyme of the kynurenine pathway [30]. IDO1 and IDO2 share a significant identity at the amino acid level but are not structurally related to TDO. IDO2 is predominantly expressed in the kidneys, followed by the epididymis, testis, and liver [31]. The expression in distinct cell types as well as the response to stimuli differs, suggesting that IDO1 and IDO2 are not functionally redundant [31]. IDO2 has even been found to be expressed in human tumor cells, but the physiologically significant degradation of TRP to KYN is catalyzed by IDO1 [32]. At present, the biological function of IDO2 is not well established. Recent studies indicate that IDO2 is a critical mediator in the production of autoantibodies [33] and in IDO1-mediated T cell regulation in the context of inflammation [34]. The existence of three rate-limiting enzymes differing in structure, substrate specificity, and activation supports the significance of the kynurenine pathway for both nutritional and immunoregulatory functions. An overview of the TRP degradation pathways and their biological significance is presented in Figure 1.

## 3. Regulation of LPS-Induced Expression of IDO1

**Figure 2:**
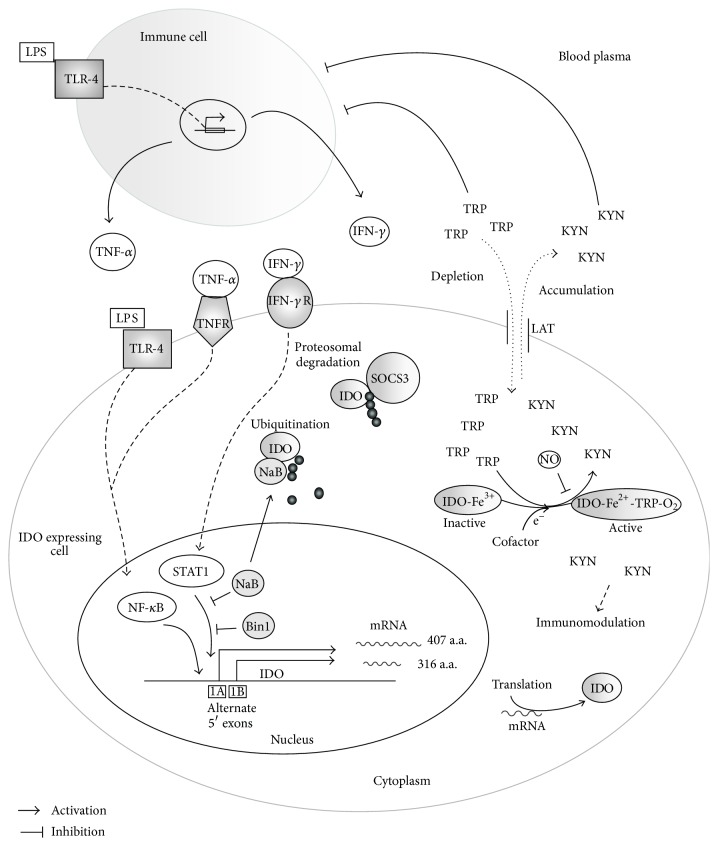
Schematic illustration of an inflammatory IDO activation by LPS and inflammatory cytokines and possible regulatory mechanisms on transcriptional, posttranscriptional, and posttranslational level.

The ubiquitously expressed intracellular enzyme IDO is an oxidoreductase and catalyzes the degradation of L-TRP to N-formylkynurenine, which, under physiologic conditions, is rapidly converted to KYN [36]. Thereby, L-TRP and KYN are transported bidirectionally by the L-amino acid transporter (LAT) to the IDO expressing cell or to the blood plasma, respectively [36]. The activation of IDO gene (*Ido*) expression is mainly induced by proinflammatory cytokines, but a constitutive expression of IDO has also been described [27, 37, 38], indicating different physiological functions of IDO. LPS act as potential ligands for various cell types, such as peripheral mononuclear cells (PBMCs), endothelial cells, cells of smooth vascular muscles, granulocytes, or thrombocytes [39]. The activation of macrophages or monocytes by LPS initiates the release of bioactive lipids, reactive oxygen species, and cytokines including IFN-*γ*, TNF-*α*, interleukin-1 (IL-1), IL-6, IL-8, or IL-10, which induce synergistically, antagonistically, or independently acting signaling pathways [40]. The activation of immune cells by LPS is mainly mediated by toll-like receptor (TLR) 4 signaling, but also by TLR-2 [41]. Therefore, LPS interact with the soluble LPS binding protein (LPB). The LPS/LPB complex binds to the membrane protein cluster of differentiation 14 (CD14), which interacts with TLR-4 and activates nuclear factor “kappa-light-chain-enhancer” of activated B-cells (NF-*κ*B) associated signaling pathways [42, 43]. The transcription factor NF-*κ*B controls more than 150 target genes, which are essential for the expression of cytokines, immune receptors, antigen-presenting molecules, proteins of acute phase, and regulatory proteins, such as transcription factors or enzymes [44]. In addition to NF-*κ*B associated IDO activation, IFN-*γ* initiates the transcription of IDO through the pathway of a signal transducer and activator of transcription 1 (STAT1). In human cancer cells, it has been shown that IDO sustains its own expression via an autocrine AhR-IL-6-STAT3 signaling loop leading to a constitutive IDO expression in IDO positive tumor cells [45].* Ido* genes have been identified in various species of mammals [46]. In mice and humans,* Ido *is located on chromosome eight and includes ten exons with approximately 15 kbp of DNA [44]. The human* Ido* gene contains multiple promoter elements and the cDNA encodes a full-length protein containing 403 amino acids with a molecular weight of 45 kDa [27]. In addition, a truncated IDO1 variant, composed of 316 amino acids, has been described [30]. The expression and activity of the IDO enzyme is complex and is regulated on transcriptional, posttranscriptional, and posttranslational levels. On the transcriptional level, IFN-*γ*-induced IDO expression is controlled by the widely expressed adaptor protein bridging integrator 1 (BIN1) or the enteral fatty acid sodium butyrate (NaB). A reduced expression or activity of BIN1, found in various types of cancers, enables an increased IDO activity, which promotes the appearance of immunotolerance [47–49].* In vitro* studies of carcinoma cells demonstrated that NaB inhibits IFN-*γ*-induced IDO expression by reducing the phosphorylation and nuclear translocation of STAT1 [50]. The IDO enzyme is a monomeric protein containing a prosthetic heme group. The catalytic activity of IDO is posttranscriptionally regulated by the intracellular redox status, indicating the existence of an IDO-specific reducing system similar to that of erythrocytes [25, 51, 52]. The oxidized enzyme (IDO-Fe^3+^) represents the catalytic inactive ferrous form, whereas the reduced ferric form of the enzyme (IDO-Fe^2+^) is catalytically active and enables the binding of TRP and oxygen [53]. Furthermore, there is evidence for NaB-dependent ubiquitination of IDO as the preparation for proteasomal degradation [54]. In the context of inflammatory immune response, the release of IFN-*γ* induces the generation of nitric-oxide (NO) by inducible nitric-oxide synthase (iNOS) [55]. NO is able to inhibit IDO activity in a reversible manner by building IDO^2+^NO-TRP complex and preventing oxidation of TRP [56]. Furthermore, NO induces the degradation of IDO via mediating the binding of suppressor of cytokine signaling 3 (SOCS3) at a specific phosphotyrosine [57, 58]. An overview of the regulation mechanisms in response to inflammatory stimuli, such as LPS, is given by Figure 2.

## 4. The Kynurenine Pathway in Endotoxemia and Sepsis

Dependent on dose and application, endotoxins, including LPS, induce the activation of inflammatory pathways, which can lead to serious systemic reactions, such as septic shock [59]. In animal models, the* in vivo* application of LPS is often used to simulate inflammatory conditions including systemic inflammatory response syndrome (SIRS), bacterial infection [60], or systemic inflammation [61]. Although the LPS-mediated activation of proinflammatory cytokines is essential for an adequate host defense, an overreaction of the proinflammatory immune response provokes serious tissue damage leading to life-threatening situations including septic shock [62]. In addition to the release of IL-10 [63], the activation of IDO is one potent immunosuppressive and regulatory mechanism of innate immunity. It has been shown that IDO expression in dendritic cells or macrophages suppresses T cells by depriving TRP [49, 64]. Additionally, it has been shown that KYN inhibits T cell-mediated inflammation by suppressing T cell proliferation [65]. Studies of human malignancies have found that IDO is highly expressed in many malignant tumor cells, leading to an effective immune escape by inhibiting the T cell response [47]. Furthermore, the generation of immunomodulating metabolites of the kynurenine pathway induced the arrest of T cells and the arrest or death of natural killer cells leading to the development of tolerogenic antigen-presenting cells (APCs) migrating to lymph nodes and mediating suppressive effects [14]. In humans, it has been shown that plasma concentrations of KYN are correlated with sepsis severity, demonstrating increased IDO activity in patients with septic shock [6]. The ratio of KYN to TRP is a sensitive marker for IDO activation and a significant increase can be used as a prognostic indicator for mortality in sepsis or trauma patients [6, 66]. Interestingly, KYN is associated with the degree of hypotension in experimental human endotoxemia [67], indicating that there are functions of IDO activation other than in immunosuppression. This is supported by studies of IDO knockout mice and mice treated with the IDO inhibitor 1-methyltryptophan. After IDO knockout or inhibition, the mice showed decreased levels of proinflammatory TNF-*α*, IL-6, and IL-12 but increased levels of the anti-inflammatory cytokine IL-10, which prevented them from dying from septic shock 48 h after the LPS challenge [68]. Furthermore, in this study, the balance of IL-12 to IL-10 had an impact on IDO protein expression, whereby IL-10 has suppressive effects on IDO protein expression if IL-10 is much higher than IL-12. It is possible that the effects of IDO induced increased hypotension or a disturbance of the balance between pro- and anti-inflammatory cytokine response. These are risk factors for mortality in early sepsis independent of immunosuppressive effects. Studies of patients with septic shock and acute kidney injury demonstrated that a failed reduction of KYNA after hemofiltration treatment may predict fatal outcomes [69]. It has been shown that KYNA* in vitro* inhibited the secretion of TNF-*α* by LPS treated mononuclear cells [70, 71]. This was confirmed by studies of mice indicating that the* in vivo* application of KYNA provokes a reduced release of TNF-*α* in response to an* ex vivo* LPS challenge [71]. Furthermore, in an* in vitro* vascular flow model, KYNA triggered the firm arrest of monocytes to both fibronectin and intercellular adhesion molecule 1 (ICAM-1), via *β*1 integrin- and *β*2 integrin-mediated mechanisms, respectively [72]. Notably, elevated plasma levels of KYNA have been reported in patients with inflammatory bowel diseases, but tryptophan and metabolites, such as 3-hydroxykynurenine, 3-hydroxyanthranilic acid, and xanthurenic acid were unchanged in all patients [73].* In vitro* studies have also provided that KYNA acts as a ligand for the G protein-coupled receptor, GPR35, that is highly expressed in human peripheral monocytes and in cells of the gastrointestinal tract [70]. However, the involvement of GPR35 in gastrointestinal disorders and its immunosuppressive effects remain unclear.

## 5. IDO-AhR-Mediated Immunosuppressive Effects on Endotoxin Tolerance and Cancer

**Figure 3:**
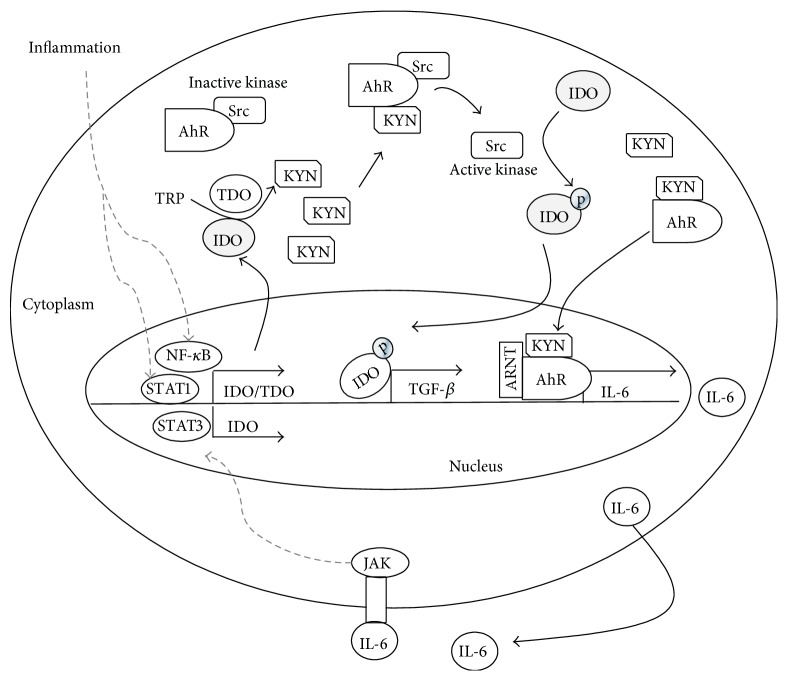
Schematic illustration of possible interaction between inflammatory IDO activation and AhR-mediated effects. IDO1 plays a crucial role in the generation of LPS-induced endotoxin tolerance whereas combined effects of AhR, IDO1, and TGF-*β* are required. L-kynurenine alone failed to restore tolerance in the absence of IDO1 indicating nonenzymatic functions as intracellular signaling. It was found that IDO1 specific phosphorylation by AhR triggered kinase activity induces reprograming of gene expression leading to TGF-*β* in response to TLR signaling. Studies on human cancer cells showed that IDO induced KYN activates the AhR-ARNT associated transcription of IL-6 which promotes autocrine activation of IDO1 via STAT3 supporting the theory that IDO-mediated immunosuppression enables effectively immune escape of tumor cells.

Endotoxin tolerance* in vivo* is characterized by a reduced inducibility of inflammatory cytokines and the HPA axis and a reduced development of illness symptoms including fever or weight loss in response to a repeated endotoxin challenge [74, 75]. In the context of endotoxin-induced sepsis, the hyperinflammatory immune response is followed by a compensatory hypoinflammatory phase up to a complete paralysis of the immune system [76], which is characterized by an endotoxin tolerance. In this phase, the proper immune response failed, leading to secondary infections (e.g., ventilator-associated pneumonia) [77]. In addition to sepsis, endotoxin tolerance has been reported in several diseases including trauma, surgery, or pancreatitis. Recent studies on IDO knockout mice indicated that IDO1 plays a crucial role in the generation of LPS-induced endotoxin tolerance and the combined effects of AhR, IDO1, and transforming growth factor-*β* (TGF-*β*) are required [78]. Notably, L-kynurenine alone failed to restore tolerance in the absence of IDO1, which indicates that nonenzymatic functions, such as intracellular signaling, are involved in the development of a regulatory phenotype in splenic dendritic cells. It has also been found that IDO1 specific phosphorylation reprograms gene expression leading to TGF-*β* production in response to TLR signaling [79]. Interestingly, the phosphorylation of IDO1 in response to LPS is dependent on the upregulation of AhR [78], which triggers the activity of Src kinase [80] resulting in phosphorylation of the target proteins [81]. The importance of AhR activation and TGF-*β* for the inducement of counterregulatory mechanisms, such as LPS tolerance, is supported by previous studies on mice indicating that the generation of regulatory T cells (T_regs_) is dependent on AhR and TGF-*β* [82, 83]. Notably, the suppressive activity of human T cells treated with TGF-1*β* plus TCDD as AhR ligand is greater than that of T cells treated only with TCDD [83]. The increase of T_regs_ is deleterious in sepsis patients and associated with decreased proliferation of effector T cells [84]. Participation of AhR in the transcriptional regulation of LPS-induced gene expression is supported by further studies showing multiple AhR associated pathways [85–87]. It has been shown that AHR-deficient mice are more sensitive to endotoxin shock than wild type mice indicating the crucial role of AhR in modulating the TLR-4-induced inflammatory immune response [88]. It is possible that the lack of AhR in these animals prevents the successful establishment or maintenance of endotoxin tolerance as a counterregulatory mechanism in response to an endotoxin challenge. AhR is a ligand-dependent cytosolic transcription factor that is able to translocate to the cell nucleus after ligand binding [86]. The tryptophan metabolites L-kynurenine [78], 6-formylindolcarbazole (FICZ, a photoproduct of TRP) [89], and KYNA [90] are described as natural AhR ligands mediating immunosuppressive functions. To induce transcription of AhR target genes in the nucleus, AhR partners with proteins such as AhR nuclear translocator (ARNT) or NF-*κ*B subunit RelB. Studies on human cancer cells have shown that KYN activates the AhR-ARNT associated transcription of IL-6, which induced autocrine activation of IDO1 via STAT3. This AhR-IL-6-STAT3 loop is associated with a poor prognosis in lung cancer [45], supporting the idea that IDO-mediated immunosuppression enables the immune escape of tumor cells. Notably, in trauma patients with increased IDO activity, nonsurvivors had higher concentrations of IL-6 after trauma than survivors [91]. It is possible that the autocrine constitutive activation of IDO is associated with immune dysfunctions and poor survival. The partnership of AhR and RelB has been shown to induce expression of IDO1 and IDO2 and to promote maturation of DCs promoting local tolerance at mucosal sites, such as the lungs or gut [86]. The prominence of AhR in immunosuppression and endotoxin tolerance may be a link between similar mechanisms found in inflammation and carcinogenesis. Potential connections between inflammatory IDO activation and AhR-mediated immunosuppressive pathways are shown in Figure 3.

However, to ensure a successful defense against pathogens, LPS tolerance is not a global downregulation of signaling proteins but is rather a mechanism of host defense that remains active [74]. In endotoxin-tolerant mice, increased numbers of Kupffer cells were detected leading to increased phagocytosis of bacteria [92, 93]. Furthermore, endotoxin-tolerant mice have an increased number of neutrophils, indicating the increased recruitment of cells from bone marrow [92] or reduced apoptosis [94]. In pigeons, a reduction of leukocytes and eosinophil granulocytes concurs with an increase in lymphocytes [95]. Even the composition of lymphocytes was changed in tolerant animals, whereas the percentage of cytotoxic T cells increased, but the percentage of T-helper cells remained unaltered. This would suggest that endotoxin tolerance helps to control inflammatory-mediated effects of bacterial infection. It was found that endotoxin-tolerant state protects mice against immunopathology in Gram-negative and Gram-positive infections [74]. Furthermore, in these studies endotoxin tolerance does not prevent the early events in protective TLR-2 signaling but prohibits the later onset of host's control over local or systemic disease.

## 6. From Immunosuppression to Immunoparalysis: The Significance of Enhanced IDO Activation

**Figure 4:**
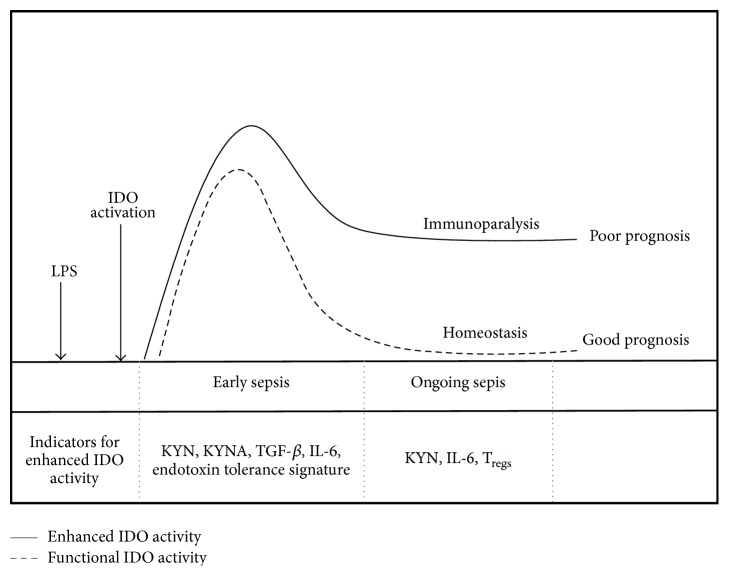
Enhanced IDO activity and its indicators as potential risk factors for the development of immunoparalysis and poor prognosis in sepsis. Patients with poor outcome have enhanced IDO activity in early state of sepsis leading to increased levels of KYN and KYNA that provokes expression of TGF-*β*, IL-6, and development of endotoxin tolerance. In ongoing sepsis, IL-6 does not decrease significantly in plasma of nonsurvivors enabling the autocrine activation of IDO via AhR-IL-6-STAT3 loop. The enhanced IDO activity mediates immunosuppressive effects as increased generation of T_regs_ in ongoing sepsis, provoking the establishment of immunoparalysis.

The mechanisms that cause the development of immunoparalysis are poorly understood. However, it has been shown that the concept of endotoxin tolerance serves as an experimental analogue to the clinical entity of immunoparalysis [96]. Microarray studies of human PBMCs showed that the expression profile of sepsis patients is strongly associated with the signature for endotoxin tolerance. Furthermore, in early stage of sepsis, the risk for the development of a confirmed sepsis or organ dysfunction was predicted by this endotoxin tolerance signature [97]. The development of immunoparalysis is strongly associated with a poor outcome in patients because the homeostasis between pro- and anti-inflammatory immune response is not restored within a few days [96]. Therefore, the protracted immunosuppressive phase prohibits the adequate control of the primary infection or of secondary hospital-acquired infections with opportunistic pathogens [98]. The role of IDO activation in the development of life-threatening immunoparalysis is still poorly understood. A recent report, which examined the AhR-mediated impact of IDO activation on immunosuppressive functions, such as LPS tolerance and immune escape of cancer cells, offers new insights for a better understanding of the development of cellular dysfunctions in immunoparalysis. It has been shown that enhanced IDO activity is associated with the severity of sepsis or septic shock [6, 66, 91, 99]. The data indicate that patients with a poor outcome have enhanced IDO activity in the early stage of sepsis, leading to increased levels of KYN and KYNA, and provoking the expression of TGF-*β* and IL-6 and the development of endotoxin tolerance. Increased plasma levels of TGF-*β* and IL-6 were found in early sepsis (day 1) of nonsurvivors compared to survivors [100]. Notably, in the ongoing sepsis of nonsurvivors (day 7), IL-6 did not significantly decrease in plasma, enabling the autocrine activation of IDO via the AhR-IL-6-STAT3 loop as described in human cells [45]. The theory of enhanced IDO activity and immunosuppression in ongoing sepsis is supported by data from patients with severe sepsis and septic shock. There, nonsurvivors had elevated KYN/TRP ratios compared to survivors, even on day 7 of sepsis, indicating enhanced IDO activity [6]. Notably, in patients with sepsis-associated immunosuppression, the treatment with granulocyte-macrophage colony-stimulating factor (GM-CSF) reduced IDO activity and downstream metabolites significantly [101]. GM-CSF is known to restore the immunocompetence of monocytes in sepsis and may shorten the time of both intrahospital and intensive care unit stay [102]. These data support that enhanced IDO activity is one of several risk factors in poor prognosis of sepsis. An illustration of risk factors detected in early and ongoing sepsis that indicates significance of enhanced IDO activity for poor outcome is given in Figure 4.

At present, there are no data regarding the IDO gene or protein expression from early to ongoing sepsis. IDO activity is often measured indirectly by the KYN/TRP ratio but TRP is also depleted by TDO and IDO2. Studies on pigs have provided LPS-inducible IDO1 protein expression in the plasma 3 h and 6 h after the LPS challenge comparable with an increase of the metabolites KYN and KYNA [61]. Even in liver and lung tissue, IDO1 protein expression was detectable 6 h after LPS. One day after the LPS challenge, in both plasma and tissue, IDO1 protein expression was not detectable in LPS-challenged pigs in accordance with the normalization of pro- and anti-inflammatory cytokines and TRP metabolites. A second LPS challenge 24 h after the first challenge did not induce cytokine production indicating LPS tolerance. However, IDO1 activation was induced neither* in vivo* nor* ex vivo *[103] suggesting that constitutive IDO1 activity is not required for maintenance of the tolerant state. It is possible that the initial phase of IDO activation is critical for a good or poor outcome after septic shock.

## 7. Conclusion

The IDO-mediated degradation of TRP along the kynurenine pathway plays a significant role as counterregulatory mechanism within the inflammatory immune response. Endotoxin-induced IDO activation results in the depletion of TRP and the generation of biological active TRP metabolites such as KYN and KYNA which have important immunosuppressive properties. Furthermore, IDO-mediated effects are required for the establishment of LPS tolerance as anti-inflammatory mechanism permitting an adequate host defense. However, an imbalance in the cytokine activation and kynurenine cascade may result in severe immune dysfunctions as immunoparalysis. Studies of patients with sepsis found that an enhanced IDO activity was associated with an increased mortality risk. However, molecular mechanisms remained poorly understood. Recent findings of IDO-AhR-mediated immunosuppressive effects in endotoxin tolerance and cancer offer new insights for the better understanding of molecular mechanisms in sepsis. In endotoxin tolerance, the AhR activation by KYN mediates the phosphorylation of IDO, inducing reprograming of gene expression and leading to TGF-*β* production in response to TLR signaling. In cancer cells a constitutive IDO activation was associated with poor prognosis. This is provoked by immunosuppressive effects of IDO expressing tumor cells on the local environment, leading to an effective immune escape of the cancer cells. Notably, AhR also mediates the constitutive IDO expression in cancer cells. Thereby, KYN activates AhR-mediated transcription of IL-6 leading to an autocrine activation of a constitutive IDO expression. It has been shown that increased concentrations of KYN or KYNA, both ligands of AhR, and increased concentrations of TGF-*β* and IL-6 are risk factors for establishment of an immunoparalysis and for a poor prognosis of patients in early sepsis instead of recovery. In ongoing sepsis, there are indices that prolonged IDO activity supports the maintenance of a protracted immunosuppressive phase. This might be due to the generation of regulatory T cells via TGF-*β* mediated pathways and an increased production of immunosuppressive metabolites such as KYN or KYNA. Increased concentrations of IL-6, found in ongoing sepsis of patients with poor prognosis, may promote the constitutive IDO activation leading to maintenance of a life-threatening immunosuppressive phase in sepsis. The significance of IDO induced AhR-mediated effects on sepsis has still to be investigated. However, the molecular mechanisms of IDO induced immunosuppression, found in endotoxin tolerance and carcinogenesis, can offer new insights on critical pathways in sepsis-associated immunoparalysis and enable new approaches for therapeutic interventions.

## Abbreviations

3-HA: 3-Hydroxyanthranilic acid
5-HT: Serotonin (5-hydroxytryptamine)
AA: Anthranilic acid
AhR: Aryl hydrocarbon receptor
APC: Antigen-presenting cell
ARNT: Aryl hydrocarbon receptor nuclear translocator
BIN1: Bridging integrator 1
CD: Cluster of differentiation
GM-CSF: Granulocyte-macrophage colony-stimulating factor
GPR35: G protein-coupled receptor
HPA: Hypothalamic-pituitary-adrenal
IDO: Indolamine 2,3-dioxygenase
IFN: Interferon
IL: Interleukin
iNOS: Inducible nitric-oxide synthase
KYN: Kynurenine
KYNA: Kynurenic acid
LBP: LPS binding protein
LPS: Lipopolysaccharides
NaB: Sodium butyrate
NAD^+^: Coenzyme nicotinamide adenine dinucleotide
NF-*κ*B: Nuclear factor “kappa-light-chain-enhancer” of activated B-cells
NO: Nitric-oxide
PBMC: Peripheral mononuclear cell
QUIN: Quinolinic acid
RelB: Subunit of the NF-*κ*B complex
SIRS: Systemic inflammatory response syndrome
SOCS3: Suppressor of cytokine signaling 3
STAT: Signal transducer and activator of transcription
TCDD: 2,3,7,8-Tetrachlorodibenzo-p-dioxin
TDO: Tryptophan 2,3-dioxygenase
TLR: Toll-like receptor
TNF: Tumor necrosis factor
TNFR: TNF receptor
TGF: Transforming growth factor
T_reg_: Regulatory T cell
TRP: Tryptophan
XA: Xanthurenic acid (3-hydroxykynurenic acid).
